# Changes in Polyphenols and Antioxidant Activity in Fermentation Substrate during Maotai-Flavored Liquor Processing

**DOI:** 10.3390/foods13121928

**Published:** 2024-06-19

**Authors:** Derang Ni, Chao Chen, Yubo Yang, Jinhu Tian, Huabin Tu, Fan Yang, Xingqian Ye

**Affiliations:** 1 Moutai Group, Institute of Science and Technology, Zunyi 564501, China; derangni@163.com (D.N.); chenchao0825@163.com (C.C.); yangyubo1207@163.com (Y.Y.); tumoutai2023@163.com (H.T.); 2 National-Local Joint Engineering Laboratory of Intelligent Food Technology and Equipment, Zhejiang Key Laboratory for Agro-Food Processing, Zhejiang Engineering Laboratory of Food Technology and Equipment, College of Biosystems Engineering and Food Science, Zhejiang University, Hangzhou 310058, China; jinhutian@126.com; 3 Zhejiang University Zhongyuan Institute, Zhengzhou 450001, China

**Keywords:** Maotai-flavored liquor, fermentation substrate, polyphenols, antioxidant activity

## Abstract

To investigate the changes in phenols and antioxidant capacity in fermented grains during different stages of the fermentation process (Xiasha, Zaosha, and single-round stages) of Maotai-flavored liquor, the total phenolic contents of 61 samples, collected in different stages, were analyzed via the Folin–Ciocalteu method, and the phenolic compounds were then identified by high-performance liquid chromatography (HPLC). Subsequently, the antioxidant activities were determined using the DPPH free radical scavenging rate and ABTS and FRAP antioxidant capacities. The correlations among the total phenolic contents, individual phenolics, and three antioxidant activities of the samples were analyzed. The results show that the total phenolic contents of the fermented samples did not change significantly in the Xiasha and Zaosha stages but showed an upward trend in the single-round stage. A total of 12 phenol acids were identified in the fermented grains, including 5 phenolic acids (e.g., ferulic acid and caffeic acid), 4 flavonoids (e.g., luteolin and apigenin), and 3 proanthocyanidins (e.g., apigeninidin), for which the DPPH free radical scavenging rates and ABTS and FRAP antioxidant capacities of all of the fermented grain samples ranged from 78.91 ± 4.09 to 98.57 ± 1.52%, 3.23 ± 0.72 to 13.69 ± 1.40 mM Trolox, and 5.06 ± 0.36 to 14.10 ± 0.69 mM FeSO_4_, respectively. The total phenolic contents of the fermented grain samples were significantly and positively correlated with the ABTS and FRAP (*p* ≤ 0.05), while no significant correlations were found between total phenolic content and DPPH. In general, the total phenolic content, phenolic substances, and antioxidant capacity of the fermented grains exhibited changes during the fermentation process in liquor production, and the phenolic components contributed more to the antioxidant properties of the fermented grains. The present study provides a theoretical reference for analyzing the dynamic changes and antioxidant properties of functional phenolic components in fermented grains.

## 1. Introduction

China has a long history of making liquors, among which Maotai-flavored liquor has a unique quality and flavor, and it is gradually becoming favored by an increasing number of people [1]. The brewing process for Maotai-flavored liquor includes two stages of grain-feeding fermentation, making sand, and seven stages of wine extraction. Fermented grains are grains that undergo steaming and fermentation and can be distilled into wine. The physical and chemical properties of fermented grains and their microorganisms change, to a certain extent, from raw material control, accumulation, and fermentation to entering and exiting the cellar [2].

Phenolic substances in fermented grains are mainly derived from the process of brewing raw materials, and the raw materials for distilling liquor mainly include wheat and sorghum [3]. Studies have shown that an abundance of phenolic substances (e.g., ferulic acid, caffeic acid, syringic acid, quercetin, and dihydro-quercetin) are found in wheat [4,5]. These phenolic substances in wheat not only affect the activity of microorganisms during the fermentation process but also generate new active ingredients through structural transformations. For example, ferulic acid can produce 4-methyl guaiacol, 4-ethyl guaiacol, and other phenolic substances, which play important roles in the flavor and taste of wine during the process of wine fermentation [6]. The phenolic substances of sorghum also have a great impact on wine quality, and the tannins in sorghum undergo enzymatic reactions, catalytic reaction, and decarboxylation during the boiling and fermentation processes, producing eugenic acid and other aromatic substances. Red tasseled sorghum is the most commonly used sorghum variety in the brewing of Maotai-flavored liquor, attributed to its suitable tannin content and amylopectin content, as well as high liquor yield [7,8]. Therefore, the identification of phenolic substances that are generated during the brewing of raw materials for Maotai-flavored liquor and the changes in their contents might provide scientific support for a standardized system to optimize the production of Baijiu.

In addition, there is a great correlation between phenolic substances in the raw materials of liquors and antioxidant capacity. Yao et al. [9] discovered ferulic acid with antioxidant activity during the brewing process for Wuliangye’s Daqu. Shi et al. [10,11] measured the in vitro antioxidant properties of five different aroma liquors, confirming their ability to scavenge free radicals, to a certain extent, and it is speculated that phenolic substances from raw materials are the key elements that impart antioxidant activity to wine. Another study found that liquor grains are rich in phenolic substances, including phenolic acids, flavonoids, and proanthocyanidins [12]. These phenolic substances exhibit high antioxidant capacities and are beneficial to human health. Therefore, measuring the antioxidant properties of the fermented grains used in Maotai-flavored liquor and establishing their relationship with phenolic substances will help in clarifying the functional components and improving the utilization efficiency of phenolic substances in fermented grains.

At present, research on phenolic substances in liquor mainly focuses on qualitative and quantitative analyses of phenolic components in liquor. Bi et al. [1] identified seven volatile phenolic compounds in Maotai-flavored liquor using the GC-MS/MS method and the contents’ ranges were determined. Hong et al. [13] detected three main phenolic substances (vanillin, 4-methylguaiacol, etc.) in liquor and confirmed their concentrations by GC-MS. However, there are relatively few studies on the identification of phenolic substances in fermented grains, and few teams have explored the changes in phenolic components in fermented grains at different sampling points and their correlations with antioxidant capacity. The present study selected wine grains sampled from 61 different nodes in the Xiasha, Zaosha, and single-round stages to explore their phenolic substances and antioxidant properties and clarify their changing patterns to improve the comprehensive utilization of wine grains and promote the sauce aroma, which can provide new insights into the healthy development of the liquor industry.

## 2. Materials and Methods

### 2.1. Materials

**Figure 1:**
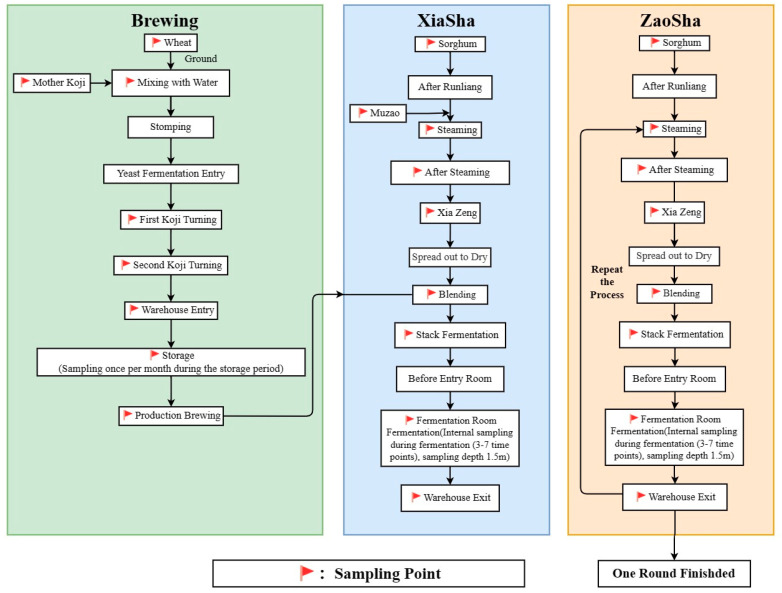
The flowchart of the process of the experiment.

Fermented grains, labeled as B1~B27-5, were provided by Kweichow Moutai Distillery (Group) Co., Ltd (Zunyi, China). The samples were taken at different time points in the Zaosha and Xiasha stages and at different depths during the single stage of accumulation fermentation (Figure 1). The main process of brewing Maotai wine includes koji making, Xiasha, Zaosha, cooking, and fermentation. Acetonitrile (pure chromatographic) and formic acid (pure-grade chromatographic) were purchased from Sigma-Aldrich Company (Milwaukee, WI, USA). Ethanol, methanol, and sodium carbonate were of analytical grade and purchased from Sinopsin Group Chemical Reagent Co., Ltd. (Shanghai, China). Folin–Ciocalteu reagent was purchased from Shanghai Maclin Biochemical Technology Co., Ltd. (Shanghai, China). Different kinds of chromatographic standards such as naringenin, apigenin, taxifolin, protocatechuic acid, and ferulic acid were purchased from Shanghai Yuanye Biotechnology Co., Ltd. (Shanghai, China). 1, 1-diphenyl-2-picrohydrazine free radical (DPPH) was purchased from Shanghai Tixiai Chemical Industry Development Co., Ltd. (Shanghai, China). Total antioxidant capacity assay kit (ABTS method) and total antioxidant capacity assay kit (FRAP method) were purchased from Shanghai Biyuntian Biotechnology Co., Ltd. (Shanghai, China).

### 2.2. Sample Preparation

Samples were taken at different time points during the Zaosha and Xiasha stages, and also at different depths during one stage of accumulation fermentation. The flow chart of the experiment is shown in Figure 1. Xiasha refers to the first infusion before the brewing begins; Zaosha refers to taking half of the raw sand, taking half of the cooked sand fermented in the first round, and mixing and distilling to start the second round of operation; one round refers to the first wine taken after two feedings and two fermentations. The samples were freeze-dried for 48 h, ground using a mill, and then sieved through an 80-mesh screen. The powder was sealed and stored at 4 °C. In total, 2 g of raw material powder was accurately weighed, added to 40 mL (solid–liquid ratio 1:20) 80% ethanol, and stirred with magnetic stirring at 75 °C for 30 min. After extraction, the liquid was filtered with a Brinell funnel, then centrifuged at 1260× *g* for 10 min, and the supernatant was removed for preservation. This step was repeated three times, and the supernatant was placed in a rotary evaporator set at 37 °C. The organic reagent was steamed under vacuum to obtain an aqueous liquid with a higher concentration.

### 2.3. Total Phenolic Determination 

The total phenolic contents were analyzed using the Folin–Ciocalteu method. Briefly, 0.1 mL sample and 0.5 mL of Folin–Ciocalteu reagent were mixed thoroughly. After 3 min, 1 mL of 7% sodium carbonate solution was added and the volume with distilled water to 10 mL, shaken well using tin foil to avoid light, and allowed to stand in a water bath at 30 °C for 2 h. The absorbance was measured at 765 nm by absorbing 200 μL liquid into the cell plate, and the results were expressed as gallic acid equivalent (mg GAE/100 g).

### 2.4. Phenolic Compound Analysis 

After rotation evaporation, the extract was added with the appropriate amount of methanol, and filtered by 0.22 μm filter membrane, and 10 μL sample was injected in a high-performance liquid chromatography system with reversed phase C-18 column (ODS C-18 column) for chromatographic separation. Two mobile phases were used for gradient elution. Phase A comprised 0.1% formic acid solution and phase B comprised pure acetonitrile. The specific elution procedure was as follows: 95–85% A, 0–5 min; 85–50% A, 5–45 min; 50–30% A, 45–47 min; 30–0% A, 47–48 min; 0% A, 48–55 min; 0–95% A, 55–56 min; 95% A, 56–65 min. The flow rate was 1.0 mL/min, the column temperature was 25 °C, and the detection wavelengths were 288 nm and 330 nm, respectively. A standard solution with concentrations of 0.01, 0.05, 0.1, 0.2, 0.4, and 0.8 mg/mL was prepared, and standard curves were drawn according to the concentration and the corresponding peak area. The standard substances used were protocatechuic acid, caffeic acid, taxifolin, p-coumaric acid, ferulic acid, and luteolin. 

### 2.5. Determination of the DPPH Free Radical Clearance

To determine the DPPH free radical clearance, 0.01 mL sample and 0.15 mL DPPH ethanol solution (0.25 g/mL) were added to the 96-well plate, mixed with vortex oscillations, incubated for 35 min in darkness, and absorbance was determined at 517 nm. A blank control group and a sample background control group were set up, with 3 parallel sets for each sample.

(1) $$\mathrm{DPPH}\;\mathrm{free}\;\mathrm{radical}\;\mathrm{clearance}\;(\%)\;=\;(1-\frac{A1-A2}{A0})\;\times \;100$$

where absorption value of A0—blank control group; A1—light absorption value of sample solution; A2—light absorption value of sample background control group.

### 2.6. ABTS Measurement

An appropriate amount of ABTS working liquid was prepared and diluted 50 times to make the absorbance of ABTS working liquid at 734 nm 0.7 ± 0.05 after subtracting the corresponding blank value, and the sample was diluted in 1.3.2 12 times prior to use. A standard solution of Trolox at 10 mM was prepared and diluted successively to 0.15, 0.30, 0.60, 0.90, 1.20, and 1.50 mM. In total, 200 μL ABTS working liquid was added to the 96-well plate, 10 μL distilled water was added to the blank group, 10 μL Trolox standard solution with different concentrations was added to the standard curve group, and the diluted samples were added to the test group at a volume of 10 μL and mixed thoroughly. After incubation at room temperature for 2–6 min, the absorption value was measured at 734 nm. The antioxidant capacity of the sample was calculated according to the standard curve.

### 2.7. FRAP Measurement

For the analysis of FRAP, 27.8 mg FeSO_4_·7H_2_O was accurately weighed, dissolved fully and set the volume to 1.00 mL, and then the standard concentration of ferrous sulfate with a concentration of 100 mM was prepared, and diluted successively to 0.15, 0.30, 0.60, 0.90, 1.20, and 1.50 mM. Trolox solutions of 0.15, 0.90, and 1.50 mM were also prepared. An appropriate amount of FRAP working liquid was prepared for incubation at 37 °C, and the sample was diluted in 1.3.2 by 10 times prior to use. In total, 180 μL of working liquid was added to the 96-well plate, 5 μL of distilled water was added to the blank group, 5 μL of ferrous sulfate standard solution with different concentrations was added to the standard curve group, and 5 μL of diluted samples were added to the test group, gently mixed, and with Trolox solution as the positive control. The absorption value at 593 nm was measured after incubation at 37 °C for 3–5 min. The total antioxidant capacity of each sample was calculated according to the standard curve.

### 2.8. Data Statistics and Analysis

SPSS 18.0 (IBM, Armonk, NY, USA) was used to analyze and process the data, and Origin 2023 (Origin Lab, Northampton, Massachusetts, USA) was used to draw graphs. Statistical analysis was performed using analysis of variance (ANOVA) via Duncan’s test, and the significance threshold was set at *p* < 0.05.

## 3. Results and Discussion

### 3.1. Total Phenol Analysis

**Figure 2:**
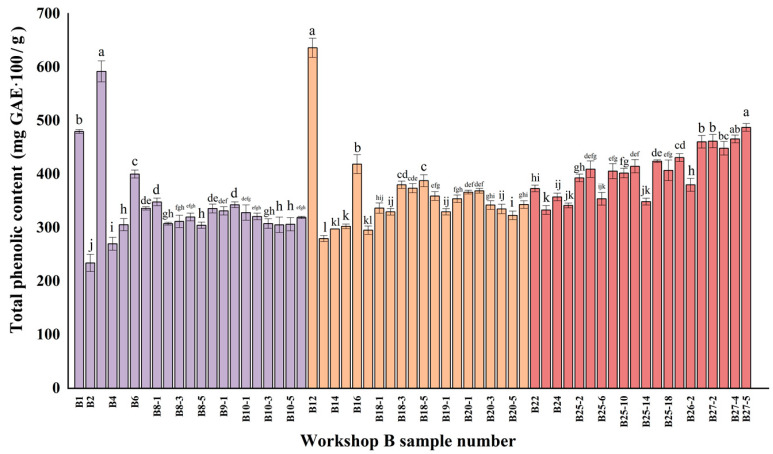
Total phenol content (mg GAE·100/g) of samples from different nodes in workshop B during Xiasha, Zaosha and single-round stages. Values in the same column with different letters are different significantly (*p* < 0.05).

Table 1 lists the names and corresponding numbers of the samples at different stages in the Xiasha, Zaosha, and one-round brewing stages, and Figure 2 directly presents the total phenol contents and changes in different samples (the difference in total phenol content in the three stages was tested for significance, *p* < 0.05). Raw sorghum samples labeled as B1 and B12 and Muzao samples (fermented grains left over from last year′s Maotai liquor brewing) numbered B3 had the highest total phenol content, reaching 475 mg GAE/100 g. The total phenol content of the fermented grains before mixing in the Muzao (B2) and grains in fermented grains before mixing in the cellar (B13) was low, accounting for half of the raw sorghum, which was speculated to be related to the high temperature and moist grains. Moistening grains are also called moistening materials, i.e., moistening the raw material (temperature ≥ 95 °C) so that the starch in the raw material absorbs water, resulting in the rupture of the starch granule cell wall [14]. High-temperature water infiltration of raw sorghum results in the loss of some water-soluble phenolic substances. Mora-Rochin et al. [15] found that the traditional cooking process would cause a significant decrease in the total phenolic content of corn raw materials (*p* < 0.05). Xiong et al. [16] reported that thermal effects could lead to the decomposition or oxidation of phenolic compounds in sorghum grains, such as *p*-coumaric acid and vanillic acid, and the stability of phenols was affected by temperature. Zhu et al. [17] found that after the cooking temperature of brewing sorghum increased from 80 °C to 100 °C, the total phenol content decreased from 0.52 to 0.41 mg/g, which was consistent with the results of the present study.

In addition, no significant changes in the total phenol content of fermented grains during precipitation and sediment-making were observed (*p* > 0.05). The phenolic content of raw materials such as red hat sorghum was relatively low, and the oxidation induced by polyphenol oxidase in sorghum might reduce the total amount of phenolic substances, whereas some of the bound polyphenols would be released from the cell wall during the fermentation process, so the overall phenolic content remained almost unchanged. Dordevic et al. [18] confirmed that fermentation could change microbial metabolism, induce changes in grain cell wall structure, and lead to the degradation or release of bioactive substances such as phenols. In the first round, the total phenol content of the samples fluctuated and increased after accumulation and cellar fermentation. In the first round, the distilling temperature was generally approximately 30–37 °C. Under suitable environmental conditions, Saccharomyces cerevisiae, amylase, protease, and xylanase from fermented grains would participate more in the fermentation process and enhance the synthesis or mutual conversion of phenolic substances. Qiu et al. [19] reported that during the process of accumulation and fermentation in the tank (temperature 23–55 °C), microorganisms continue to grow and synthesize phenols and other active substances through various biochemical reactions. After the accumulation and fermentation of raw materials in the first two stages, the damage degree of the cell wall was higher, the cell components were more decomposed, and the release of bound phenolic substances was also increased. In addition, samples with a stacking depth of 0.3 m showed a higher total phenol content than samples with a stacking depth of 1.5 m during the same time. This was related to the spatial distribution of microorganisms during accumulation fermentation. The number of microorganisms in the surface layer was higher than that in the bottom layer [20], and microbial diversity played an important role in increasing the type and content of phenolic substances. The content of phenolic compounds was increased by Xiasha, Zaosha, and one-round brewing stages. The changes in total phenol content might be the result of the interaction between microorganisms, enzymes, and sample components during the fermentation process [21].

### 3.2. Identification of Phenolic Substances

**Figure 3:**
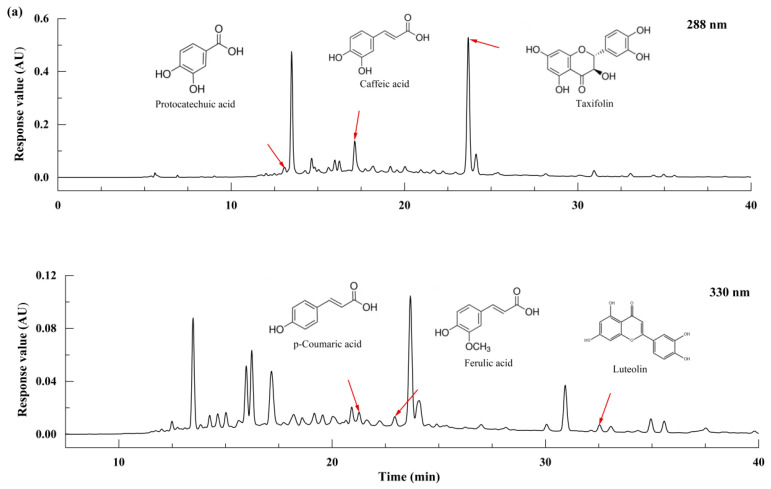

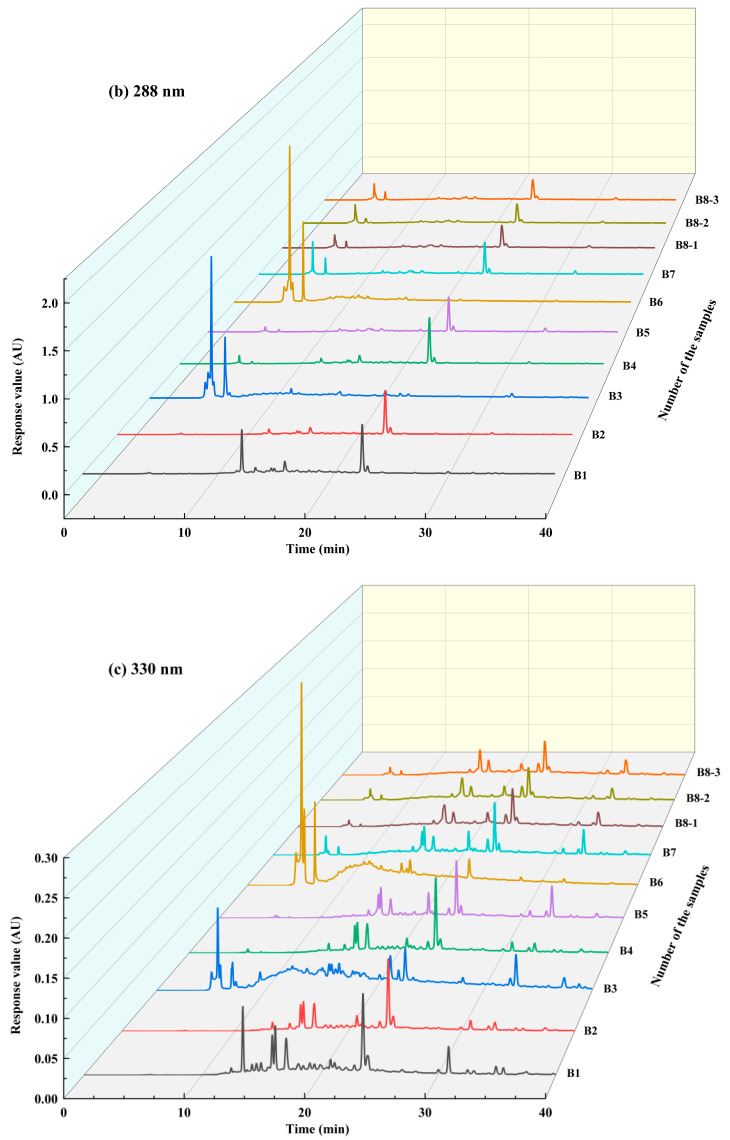
HPLC spectra of some samples from Workshop B at Xiasha, Zaosha and single-round stages. (**a**) The peaks of B1 samples at 288 and 330 nm and their correspondence with phenolics; (**b**,**c**) Comparison of the peaks of B1~B8-3 samples at 288 and 330 nm.

**Figure 4:**
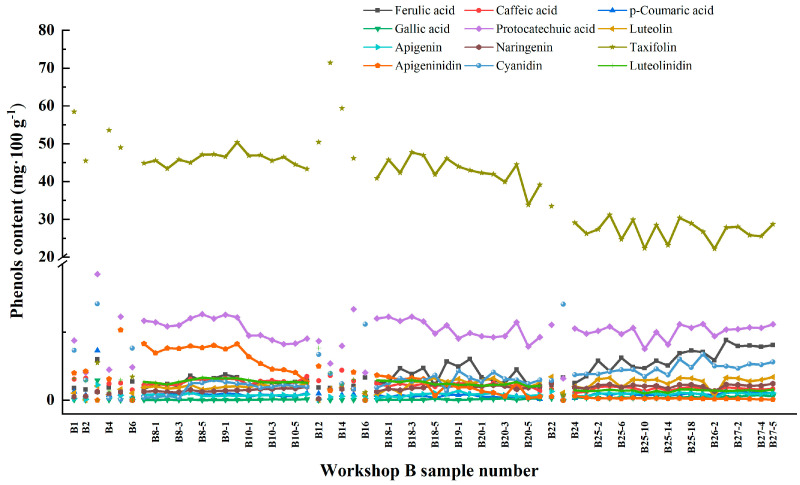
Content of 12 phenols (mg/100 g) in samples at different nodes in Workshop B at Xiasha, Zaosha and single-round stages.

Figure 3 shows the HPLC spectra of the samples at the Xiasha, Zaosha and single-round stages. Figure 4 shows the content of phenolic substances in the samples at different stages. Twelve phenolic substances were identified in all samples (Table 2), including five phenolic acids, such as caffeic acid and ferulic acid, four flavonoids such as luteolin and apigenin, and apigenin. The average content of taxifolin and protocatechuic acid was 37.518 mg/100 g of raw powder and 4.951 mg/100 g of protocatechuic acid in the three brewing stages of Xiasha, Zaosha, and single-round stages. In contrast, gallic acid had the lowest phenolic content, with an average of only 0.124 mg/100 g. Shreeya Ravisankar et al. [22] detected ferulic acid, luteolin, apigenin, and several anthocyanin isomers in sorghum raw materials. Shen et al. [23] identified 10 phenolic substances from 8 types of sorghum raw materials from different producing areas, among which the contents of taxifolin and protocatechuic acid were relatively high. In the stages of sediment lowering, sediment building and the first round, the contents of the phenolic substances changed greatly, mainly including ferulic acid, apigenin, and cyanidin, while the other eight phenolic substances did not change significantly. Among them, the content of taxifolin showed a decreasing trend (from 44.840 to 28.685 mg/100 g), and the content of celery also showed a decreasing trend in the three stages (from 4.135 to 0.044 mg/100 g). In contrast, ferulic acid and cyanidin increased from 0.835 to 4.039 mg/100 g and from 0.200 to 2.798 mg/100 g, respectively. Yan et al. [24] reported that during the fermentation of Luzhou-flavored liquor, the content of ferulic acid in the samples of fermented grains showed a trend of first decreasing and then increasing with the extension of fermentation time, which was consistent with the results of the present study.

In the process of brewing liquor, koji mixing, stacking, and cellar fermentation enrich microorganisms, including bacteria, yeasts and molds [25]. Wang et al. [26] pointed out that the first-round brewing stage was in winter, and bacteria that could adapt to lower temperatures could also grow normally, and the bacterial structure presented the characteristics of multiple species and relatively average abundance. The dominant strains included *Bacillus*, *Dieffteria*, *Sphingobacterium*, and *Pseudomonas*. There were obvious differences in the species and quantity of yeast at different stages of fermentation. *P. kudriavzevii* was the dominant yeast in the stages of sediment-sinking and sediment-making, and there were more dominant strains in the first round. *Candida apicola* and *Saccharomyces cerevisiae* were added [27]. The dominant molds in fermented grains were *Penicillium* and *Aspergillus*, which secrete cellulase, pectinase, protease, and other important enzymes. Lu et al. [28] screened an *Aspergillus niger* strain that could produce phenolic substances after solid fermentation from Daqu.

The brewing environment, the types and quantities of dominant microorganisms, and the types and activities of key enzymes may be one of the reasons for the changes in the content of some phenols in the three brewing stages. It was speculated that there were different microorganisms related to phenolic metabolisms, such as *Bacillus subtilis*, *Bacillus amyloolytica*, *Delfordia*, *Saccharomyces cerevisiae*, *Aspergillus niger*, and their enzymes in different stages of fermented grains, which caused structural transformation or mutual transformation of phenolic substances and, greatly affected the contents of taxifolin and ferulic acid in different stages.

### 3.3. Antioxidant Activity Analysis

**Figure 5:**
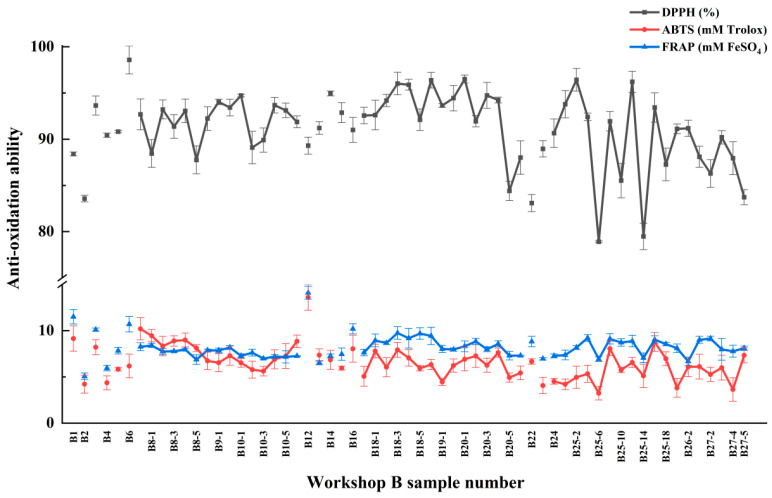
Antioxidant capacity of samples from different nodes in workshop B during Xiasha, Zaosha and single-round stages.

Table 3 and Figure 5 show the antioxidant capacity of samples at Xiasha, Zaosha, and single-round stages. The DPPH free radical scavenging rate, ABTS antioxidant capacity, and FRAP antioxidant capacity were used to evaluate the antioxidant activity of fermented grains. The results showed that the total DPPH free radical scavenging rate showed little difference, ranging from 78.91 ± 4.09 to 98.57 ± 1.52%. In the first round, the clearance rate of fermented grains with a stacking time of 3 days and a stacking depth of 1.5 m (B25-6) was the lowest, whereas the clearance rate of Daqu samples with a stacking depth of B6 was the highest. Yao et al. [9] used Daqu, a Luzhou-flavored liquor, as the research object, identified six phenolic chemical components in Daqu, and confirmed that it had a strong DPPH free radical scavenging rate: at a concentration of 25.0 μmol/L, the total DPPH scavenging rate of the six phenolic mixtures could reach 94.5 ± 0.9%. The antioxidant capacity of ABTS and FRAP of all samples ranged from 3.23 ± 0.72 to 13.69 ± 1.40 mM Trolox and 5.06 ± 0.36 to 14.10 ± 0.69 mM FeSO_4_, respectively, indicating that all samples had certain antioxidant properties. Among them, the raw sorghum sample numbered as B12 showed the strongest antioxidant capacity. The samples of stacked grains numbered B25-6 had the weakest ABTS antioxidant capacity, which was consistent with the results of DPPH above, and the samples of stacked grains numbered B2 before the mixing Muzao had the lowest FRAP antioxidant capacity. The DPPH free radical clearance rate and the antioxidant capacity of ABTS decreased from the stage of precipitation and sand-making to the first round, whereas the antioxidant capacity of FRAP did not increase or decrease significantly. In the process of stacking fermentation, samples in fermented grains were comprehensively affected by microorganisms, enzymes, ambient temperature, pH, and other ingredients in raw materials [27], resulting in changes in total phenol content and phenolic monomer content, as well as changes in the antioxidant capacity of samples.

In addition, the accumulation depth affects the oxidation resistance of the samples during the first round. When the accumulation time was the same, the antioxidant capacity of most samples with a deposition depth of 0.3 m was higher than that of samples with a deposition depth of 1.5 m, and it was also found in Section 2.1 that the samples with a shallow deposition depth had a higher total phenol content. This indicated a certain correlation between the antioxidant capacity and the total phenol content of the samples during a round of accumulation fermentation. During the stacking process of fermented grains, water content, temperature, oxygen content, microbial community structure, and other conditions of the surface layer, surface layer, and core pile with different depths showed significant differences [20]. The surface water content was slightly lower, the temperature was higher, and the oxygen content was higher, these environmental factors were conducive to the growth and reproduction of fermentation microorganisms and improved their enzyme production ability. It was speculated that microorganisms played a key role in increasing the antioxidant chemical components such as phenols, polysaccharides, vitamins, and their derivatives in the samples, and could improve the total antioxidant capacity of the samples by regulating the synthesis, degradation, or transformation of these substances, which also explained that the surface accumulation of samples with more microorganisms showed stronger antioxidant capacity.

### 3.4. Correlation Analysis

**Figure 6:**
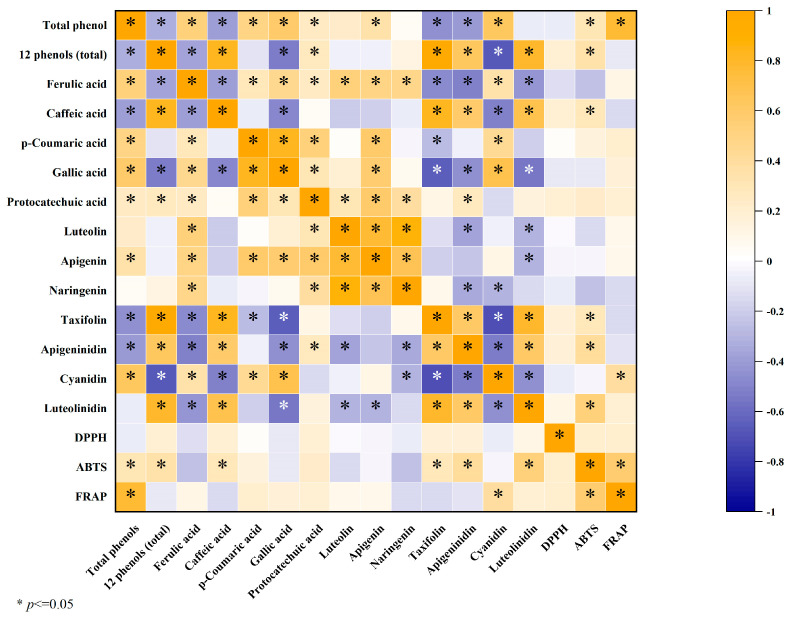
Heat map of correlation between total phenols, 12 phenolics, DPPH, ABTS and FRAP of different node samples.

By analyzing the correlation heat map (Figure 6), the following conclusions can be drawn. The total phenol content of fermented grains was significantly positively correlated with ABTS and FRAP (*p* ≤ 0.05), but not significantly correlated with DPPH. Due to the complex matrix and diverse composition of the samples, DPPH was presumed to be related to polysaccharides, vitamins, and derivatives with antioxidant activities in addition to phenols. In general, the samples of fermented grains with higher total phenol content showed stronger antioxidant capacity, which indicated that phenols had a greater contribution to the antioxidant capacity. Yang et al. [29] also showed that the antioxidant activity of soybean was directly affected by the difference in phenolic content, and the content of soybean polyphenol was significantly correlated with hydroxyl free radical clearance and iron reduction ability (*p* ≤ 0.01). Phenolic substances contain one or more aromatic rings with hydroxyl groups and form a polyhydroxyl-conjugated system, which can easily supply hydrogen from the aromatic cyclic hydroxyl group. Their strong reducibility and ability to remove free radicals made them effective antioxidants [30], and ABTS and FRAP were significantly positively correlated (*p* ≤ 0.05).

It is worth noting that the total phenol content was significantly negatively correlated with the sum of the 12 phenolic substances (*p* ≤ 0.05), suggesting that there were still some phenolic components in the fermented grains samples that were not identified under the experimental conditions. The fermented grains of the Maotai-flavored liquor were composed of wheat and sorghum as the main raw materials and Dakoji as the saccharification starter. Fermented grains were rich in phenolic acids, flavones, and proanthocyanidin. The sources of phenols are diverse, and the specific composition and content of phenols are complicated. Chetrariu et al. [12] identified a variety of phenols, such as protocatechuic acid, vanillic acid, caffeic acid, chlorogenic acid, *p*-coumaric acid, and rosmarinic acid, from discarded fermented grains. Wang et al. [31] extracted eight phenolic acids, such as trans-ferulic acid, vanillic acid and 4-hydroxybenzoic acid, from fermented grains. Yang et al. [32] confirmed that a variety of flavonoids such as flavokawain B, isosinensetin, 8-prenylnaringenin and 6-demethoxytangeretin could be identified in liquor grain extracts.

Ferulic acid was positively correlated with the contents of *p*-coumaric acid, gallic acid, protocatechuic acid, luteolin, apigenin, naringin, and cyanidin, whereas it was negatively correlated with the contents of caffeic acid, taxifolin, apigenin, and luteolin (*p* ≤ 0.05). Phenols other than ferulic acid also correlated with the remaining 11 phenols. This is consistent with the conclusion in Section 2.2 that the content of taxifolin and celery decreased while the content of ferulic acid and cyanidin increased from the stage of sediment lowering and Zaosha to the first round, which further indicated that some phenolic substances might undergo structural transformation or mutual transformation during the accumulation fermentation process.

## 4. Conclusions

The total phenol content of fermented grains did not change significantly during the stages of Xiasha and Zaosha. However, the total phenol content fluctuated and increased in the first round after the samples were accumulated and fermented in the pit. In total, 12 phenolic substances were identified in 61 fermented grains, including 5 phenolic acids (ferulic acid and caffeic acid), and 4 flavonoids such as luteolin and apigenin. The contents of taxifolin and apigenin fluctuated in three stages (from 44.840 mg/100 g to 28.685 mg/100 g, respectively). The contents of ferulic acid and cyanidin fluctuated (from 0.835 to 4.039 mg/100 g and from 0.200 to 2.798 mg/100 g, respectively). The DPPH free radical scavenging rate and the antioxidant capacity of ABTS and FRAP were 78.91 ± 4.09~98.57 ± 1.52%, 3.23 ± 0.72~13.69 ± 1.40 mM Trolox and 5.06 ± 0.36~14.10 ± 0.69 mM FeSO_4_, respectively. All samples showed antioxidant activity, and there was a significant positive correlation between total phenol content and ABTS and FRAP (*p* ≤ 0.05), but no significant correlation between total phenol content and DPPH, indicating that phenolic components contributed significantly to the antioxidant properties of fermented grains. In addition, most grains with a stacking depth of 0.3 m had higher total phenol content and stronger antioxidant capacity than those with a stacking depth of 1.5 m in one round with the same stacking time. This study provides insights for deepening the functional study of phenolic components in fermented grains and improving the comprehensive utilization rate of fermented grains in Maotai-flavored liquor. In conclusion, phenolic compounds such as protocatechuic acid, caffeic acid, taxifolin, *p*-coumaric acid, ferulic acid, and luteolin were identified in different fermentation processes of Maotai based on the extraction system and the HPLC methods.

## Figures and Tables

**Table 1:** Name and corresponding number of samples from different nodes in Workshop B at Xiasha, Zaosha and single-round stages.

Sample Name of Xiasha Stage		Number	Sample Name of Zaosha Stage		Number	Sample Name of Single-Round Stage		Number
Raw sorghum		B1	Raw sorghum		B12	Wine spirits before mixing with Daqu		B22
Pre-mixing grain spirits		B2	Grain spirits before mixing with cellar spirits		B13	Daqu		B23
Muzao		B3	Grain spirits after mixing with cellar spirits		B14	Wine spirits after mixing with Daqu		B24
Grain spirits after mixing Muzao		B4	Wine spirits before mixing with Daqu		B15	Stacking 1 day of wine spirits	Depth 0.3 m	B25-1
Wine spirits before mixing with Daqu		B5	Daqu		B16		Depth 1.5 m	B25-2
Daqu		B6	Wine spirits after mixing with Daqu		B17	Stacking 3 day of wine spirits	Depth 0.3 m	B25-5
Wine spirits after mixing with Daqu		B7	Stacking 1 day of wine spirits	Depth 0.3 m	B18-1		Depth 1.5 m	B25-6
Stacking 1 day of wine spirits	Depth 0.3 m	B8-1		Depth 1.5 m	B18-2	Stacking 5 day of wine spirits	Depth 0.3 m	B25-9
	Depth 1.5 m	B8-2	Stacking 2 day of wine spirits	Depth 0.3 m	B18-3		Depth 1.5 m	B25-10
Stacking 2 day of wine spirits	Depth 0.3 m	B8-3		Depth 1.5 m	B18-4	Stacking 7 day of wine spirits	Depth 0.3 m	B25-13
	Depth 1.5 m	B8-4	Stacking 3 day of wine spirits	Depth 0.3 m	B18-5		Depth 1.5 m	B25-14
Stacking 3 day of wine spirits	Depth 0.3 m	B8-5		Depth 1.5 m	B18-6	Stacking 9 day of wine spirits	Depth 0.3 m	B25-17
	Depth 1.5 m	B8-6	Pre-cellar spirits	Depth 0.3 m	B19-1		Depth 1.5 m	B25-18
Pre-cellar spirits	Depth 0.3 m	B9-1		Depth 1.5 m	B19-2	Pre-cellar spirits	Depth 0.3 m	B26-1
	Depth 1.5 m	B9-2	Fermentation of wine spirits in the cellar for 3 days		B20-1		Depth 1.5 m	B26-2
Fermentation of wine spirits in the cellar for 3 days		B10-1	Fermentation of wine spirits in the cellar for 7 days		B20-2	Fermentation of wine spirits in the cellar for 3 days		B27-1
Fermentation of wine spirits in the cellar for 7 days		B10-2	Fermentation of wine spirits in the cellar for 14 days		B20-3	Fermentation of wine spirits in the cellar for 7 days		B27-2
Fermentation of wine spirits in the cellar for 14 days		B10-3	Fermentation of wine spirits in the cellar for 21 days		B20-4	Fermentation of wine spirits in the cellar for 14 days		B27-3
Fermentation of wine spirits in the cellar for 21 days		B10-4	Fermentation of wine spirits in the cellar for 28 days		B20-5	Fermentation of wine spirits in the cellar for 21 days		B27-4
Fermentation of wine spirits in the cellar for 28 days		B10-5	Unstrained spirits from the cellar		B21	Fermentation of wine spirits in the cellar for 28 days		B27-5
Unstrained spirits from the cellar		B11						

**Table 2:** Chemical formulae and contents of 12 phenolic substances in sorghum “Jinhongying II”.

Phenolic Substances	Chemical Formula	Content (mg/100 g)
Gallic acid	C_7_H_6_O_5_	0.025 ± 0.003
Protocatechuic acid	C_7_H_6_O_4_	4.369 ± 0.152
Caffeic acid	C_9_H_8_O_4_	1.527 ± 0.340
Luteolinidin	C_15_H_11_O_5_^+^	1.571 ± 0.220
Cyanidin	C_15_H_11_O_6_^+^	3.650 ± 0.053
Apigeninidin	C_15_H_11_O_4_^+^	1.985 ± 0.352
*p*-Coumaric acid	C_9_H_8_O_3_	0.606 ± 0.012
Ferulic acid	C_10_H_10_O_4_	0.882 ± 0.105
Taxifolin	C_15_H_12_O_7_	58.433 ± 3.266
Luteolin	C_15_H_10_O_6_	0.512 ± 0.113
Apigenin	C_15_H_10_O_5_	0.118 ± 0.011
Naringenin	C_15_H_12_O_5_	0.246 ± 0.037

**Table 3:** DPPH radical scavenging rate, ABTS and FRAP total antioxidant capacity of samples from different nodes in Workshop B at Xiasha, Zaosha and single-round stages.

Sample Number	DPPH (%)	ABTS (mM Trolox)	FRAP (mM FeSO_4_)
B1	88.40 ± 0.21	9.15 ± 1.37	11.52 ± 0.77
B2	83.57 ± 3.36	4.22 ± 0.97	5.06 ± 0.36
B3	93.64 ± 1.03	8.21 ± 0.81	10.16 ± 0.18
B4	90.42 ± 0.23	4.36 ± 0.76	5.95 ± 0.30
B5	90.82 ± 2.14	5.83 ± 0.20	7.83 ± 0.36
B6	98.57 ± 1.52	6.18 ± 1.28	10.70 ± 0.84
B7	92.68 ± 1.67	10.24 ± 1.2	8.26 ± 0.43
B8-1	88.45 ± 3.50	9.44 ± 0.67	8.40 ± 0.21
B8-2	93.21 ± 1.02	8.33 ± 1.05	7.75 ± 0.31
B8-3	91.37 ± 1.28	8.89 ± 0.56	7.77 ± 0.10
B8-4	93.06 ± 1.28	8.98 ± 0.75	7.95 ± 0.16
B8-5	87.76 ± 4.51	8.12 ± 0.38	6.87 ± 0.52
B8-6	92.23 ± 1.28	6.73 ± 0.94	7.87 ± 0.13
B9-1	94.03 ± 0.30	6.54 ± 0.96	7.86 ± 0.26
B9-2	93.42 ± 0.91	7.29 ± 1.06	8.17 ± 0.21
B10-1	94.72 ± 0.15	6.52 ± 0.48	7.27 ± 0.22
B10-2	89.09 ± 4.76	5.78 ± 0.92	7.62 ± 0.37
B10-3	89.90 ± 3.31	5.62 ± 0.52	6.98 ± 0.08
B10-4	93.68 ± 1.84	6.90 ± 1.03	7.19 ± 0.21
B10-5	93.11 ± 1.79	7.25 ± 1.34	7.16 ± 0.66
B11	91.87 ± 0.64	8.84 ± 0.67	7.26 ± 0.05
B12	89.30 ± 0.92	13.69 ± 1.4	14.1 ± 0.69
B13	91.21 ± 0.69	7.36 ± 0.66	6.53 ± 0.22
B14	94.94 ± 0.26	6.83 ± 1.03	7.26 ± 0.21
B15	92.87 ± 1.09	5.95 ± 0.21	7.46 ± 0.67
B16	91.00 ± 1.35	8.04 ± 1.46	10.21 ± 0.55
B17	92.56 ± 1.89	5.06 ± 1.08	7.63 ± 0.35
B18-1	92.61 ± 1.60	7.79 ± 0.74	8.94 ± 0.70
B18-2	94.18 ± 1.66	6.05 ± 1.04	8.66 ± 0.17
B18-3	96.01 ± 1.21	7.92 ± 0.81	9.75 ± 0.68
B18-4	95.89 ± 0.58	7.06 ± 0.90	9.18 ± 1.06
B18-5	92.10 ± 4.16	5.93 ± 0.30	9.69 ± 0.62
B18-6	96.37 ± 2.84	6.33 ± 0.55	9.43 ± 0.93
B19-1	93.64 ± 2.20	4.46 ± 0.38	8.02 ± 0.39
B19-2	94.44 ± 2.38	6.22 ± 0.71	7.95 ± 0.18
B20-1	96.50 ± 0.43	6.91 ± 1.25	8.31 ± 0.58
B20-2	91.94 ± 2.60	7.26 ± 1.23	8.83 ± 0.33
B20-3	94.73 ± 2.41	6.26 ± 0.76	7.99 ± 0.26
B20-4	94.23 ± 0.31	7.62 ± 0.50	8.57 ± 0.33
B20-5	84.40 ± 4.04	4.93 ± 0.50	7.30 ± 0.47
B21	88.01 ± 1.81	5.43 ± 0.74	7.30 ± 0.07
B22	83.09 ± 0.92	6.66 ± 0.30	8.82 ± 0.57
B23	88.96 ± 3.89	4.06 ± 0.88	6.96 ± 0.12
B24	90.65 ± 1.53	4.52 ± 0.30	7.26 ± 0.21
B25-1	93.78 ± 1.48	4.20 ± 0.57	7.37 ± 0.52
B25-2	96.42 ± 1.23	4.95 ± 1.20	8.17 ± 0.21
B25-5	92.41 ± 0.42	5.33 ± 0.93	9.21 ± 0.36
B25-6	78.91 ± 4.09	3.23 ± 0.72	6.93 ± 0.26
B25-9	91.94 ± 1.06	8.05 ± 0.75	9.11 ± 0.55
B25-10	85.52 ± 4.87	5.75 ± 0.29	8.72 ± 0.40
B25-13	96.21 ± 1.13	6.56 ± 0.54	8.87 ± 0.62
B25-14	79.48 ± 5.43	5.11 ± 1.25	7.06 ± 0.53
B25-17	93.42 ± 2.59	8.78 ± 1.02	9.03 ± 0.41
B25-18	87.26 ± 3.76	6.96 ± 0.75	8.55 ± 0.19
B26-1	91.11 ± 3.52	3.81 ± 1.02	8.10 ± 0.46
B26-2	91.17 ± 3.88	6.08 ± 1.06	6.67 ± 0.43
B27-1	88.10 ± 2.15	6.11 ± 1.34	9.01 ± 0.38
B27-2	86.29 ± 2.50	5.26 ± 0.77	9.14 ± 0.25
B27-3	90.20 ± 0.72	5.99 ± 1.34	7.98 ± 1.17
B27-4	87.94 ± 1.78	3.64 ± 1.27	7.77 ± 0.65
B27-5	83.72 ± 1.81	7.33 ± 0.82	8.10 ± 0.24

## Data Availability

The original contributions presented in the study are included in the article, further inquiries can be directed to the corresponding authors.
